# The neuroprotective effects of normobaric oxygen therapy after stroke

**DOI:** 10.1111/cns.14858

**Published:** 2024-07-15

**Authors:** Chuan Li, Min Jiang, Zhiying Chen, Qiongqiong Hu, Ziying Liu, Junmin Wang, Xiaoping Yin, Jian Wang, Moxin Wu

**Affiliations:** ^1^ Department of Medical Laboratory Affiliated Hospital of Jiujiang University Jiujiang Jiangxi China; ^2^ Jiujiang Clinical Precision Medicine Research Center Jiujiang Jiangxi China; ^3^ Department of Neurology Affiliated Hospital of Jiujiang University Jiujiang Jiangxi China; ^4^ Department of Neurology Zhengzhou Central Hospital, Zhengzhou University Zhengzhou Henan China; ^5^ Department of Human Anatomy School of Basic Medical Sciences, Zhengzhou University Zhengzhou Henan China

**Keywords:** cerebral hypoxia, neuroprotection, normobaric hyperoxia, oxygen therapy, stroke

## Abstract

**Background:**

Stroke, including ischemic and hemorrhagic stroke, is a severe and prevalent acute cerebrovascular disease. The development of hypoxia following stroke can trigger a cascade of pathological events, including mitochondrial dysfunction, energy deficiency, oxidative stress, neuroinflammation, and excitotoxicity, all of which are often associated with unfavorable prognosis. Nonetheless, a noninvasive intervention, referred to as normobaric hyperoxia (NBO), is known to have neuroprotective effects against stroke.

**Results:**

NBO can exert neuroprotective effects through various mechanisms, such as the rescue of hypoxic tissues, preservation of the blood–brain barrier, reduction of brain edema, alleviation of neuroinflammation, improvement of mitochondrial function, mitigation of oxidative stress, reduction of excitotoxicity, and inhibition of apoptosis. These mechanisms may help improve the prognosis of stroke patients.

**Conclusions:**

This review summarizes the mechanism by which hypoxia causes brain injury and how NBO can act as a neuroprotective therapy to treat stroke. We conclude that NBO has significant potential for treating stroke and may represent a novel therapeutic strategy.

## Graphical abstract

After stroke, hypoxia causes a series of pathological events, including energy deficiency, mitochondrial dysfunction, oxidative stress, neuroinflammation, and excitoxicity. NBO treatment rescues this pathological process and has a neuroprotective effect.

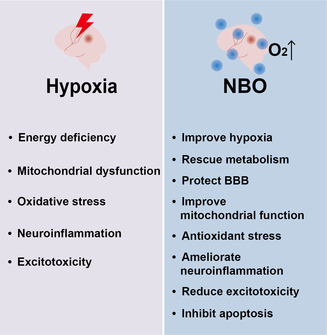

## 1 INTRODUCTION

Stroke, including ischemic stroke and hemorrhagic stroke, is a common cerebrovascular disease caused by insufficient blood supply to the brain or blood vessel rupture within the brain parenchyma.^1^, ^2^ Stroke, the second leading cause of death worldwide after cardiovascular disease, affects more than 15 million people each year.^3^ One of the hallmarks of stroke is the interruption of cerebral blood flow (CBF), which depletes the brain of oxygen and glucose. This leads to the disruption of adenosine 5′‐triphosphate (ATP) synthesis, impaired ion homeostasis, and acid–base balance. All these dysfunctions result in cerebral neuropathological changes, such as brain edema, neuroinflammation, and neuronal cell death, eventually leading to severe neurological deficits.

Molecular oxygen is an indispensable component for cellular homeostasis and survival in aerobic organisms, as it is the final receiver of electrons in the mitochondrial respiratory chain and ATP synthesis through phosphate oxidation.^4^ Brain tissue accounts for only 2% of the human body weight. It consumes more than 20% of total oxygen to generate ATP to increase the membrane potential required for energy utilization and supply.^5^ As the main energy consumer, the brain has low capillary density, is the most vulnerable organ to hypoxia, and relies on a constant oxygen supply.^6^ The normal range of partial pressure of arterial oxygen (PaO_2_) is 11.0–14.4 kPa, and the normal range of arterial oxygen saturation (SaO_2_) is 95%–98% in adults.^7^ Hypoxia is a condition in which the body or a region lacks adequate oxygen at the tissue level. Hypoxia is a common feature after acute stroke and is associated with neurological deficits and increased mortality.^8^, ^9^ Hypoxia immediately causes neuronal cell death and possibly impairs brain function. In addition, mounting evidence suggests that hypoxia affects many pathological aspects of stroke, including neuroinflammation, oxidative stress, mitochondrial impairment, and synaptic dysfunction, which collectively contribute to secondary brain injury (SBI) after stroke.

Therefore, oxygen therapy may delay or mitigate the progression of stroke. Oxygen therapy refers to a medical procedure that relieves hypoxia by increasing the PaO_2_ in the inhaled gas and SaO_2_ in the blood.^10^ It is widely used for central nervous system (CNS) disease therapy. Standard oxygen therapy includes hyperbaric oxygen (HBO) and normobaric oxygen (NBO) therapy. HBO therapy delivers oxygen at hyperbaric pressure (2.5 atm, equivalent to 253 kPa) to increase PaO_2_ in tissues. NBO therapy involves the administration of 40%–100% high‐flow oxygen under normal pressure to supplement the oxygen supply and increase the arterial oxygen content. To better understand the neuroprotective effects of NBO treatment after stroke, we briefly summarize the effects of hypoxia and NBO treatment on stroke and discuss the underlying mechanisms involved.

## 2 EVIDENCE OF HYPOXIA AFTER STROKE

Previously, Read et al.^11^ detected peri‐infarct tissue hypoxia via positron emission tomography (PET) with 18F‐fluoromisonidazole (18F‐FMISO) in ischemic stroke patients. Hypoxia was detected in the peri‐infarct tissue of 9 of the 13 patients within 48 h of stroke onset. The kinetics of intravenous bolus injection of indocyanine green (ICG) in patients with acute ischemic stroke (AIS) were monitored by near‐infrared spectroscopy (NIRS). Compared with those in the unaffected hemisphere, the bolus peak time, peak time, and rise time were increased, and the slope and blood flow index of the infarct site were reduced in the infarcted hemisphere.^12^ Lu et al.^13^ used quantitative susceptibility‐weighted imaging to more accurately determine hypoperfusion and penumbral hypoxia in patients with AIS. The serum levels of hypoxia‐inducible factor 1α (HIF‐1α), a master transcriptional regulator of the cellular response to hypoxia, were significantly elevated in stroke patients compared with healthy individuals and were positively correlated with clinical outcomes.^14^ Hypoxic tissue was observed in middle cerebral artery occlusion (MCAO) model rats by magnetic resonance imaging (MRI) and pimonidazole staining, and hypoxic areas were observed in both groups.^15^ Furthermore, tissue‐integrating oxygen sensors continuously monitor tissue oxygen reduction in rats with carotid artery occlusion.^16^

Similarly, brain tissue oxygen monitoring shows insufficient oxygen supply to brain tissue in most intracerebral hemorrhage (ICH) patients.^17^ The partial pressure of oxygen in the brain tissue of ICH patients was monitored by neuromonitoring probes and found to be significantly lower in the hemorrhagic brain tissue than in healthy tissue.^18^ Recent studies have reported that serum HIF‐1α levels are markedly higher in ICH patients than in healthy controls and are closely related to hemorrhagic severity and 90‐day outcomes.^19^ In addition, 18F‐FMISO PET/MRI imaging revealed that hypoxia is expected in the perihematomal area in collagenase VII‐induced ICH model rats and the uptake of 18F‐FMISO significantly increased 18–24 h after ICH.^20^ With hematoma formation, the increase in intracranial pressure and secondary hematoma volume further exacerbated brain tissue hypoxia in a porcine ICH model.^17^ In contrast, using 18F‐FMISO PET images, Hirano et al.^21^ showed that patients with ICH did not exhibit tissue hypoxia.

## 3 PATHOPHYSIOLOGY AND SIGNALING PATHWAYS AFFECTED BY HYPOXIA AFTER STROKE

### 3.1 Energy deficiency due to hypoxia

**FIGURE 1:**
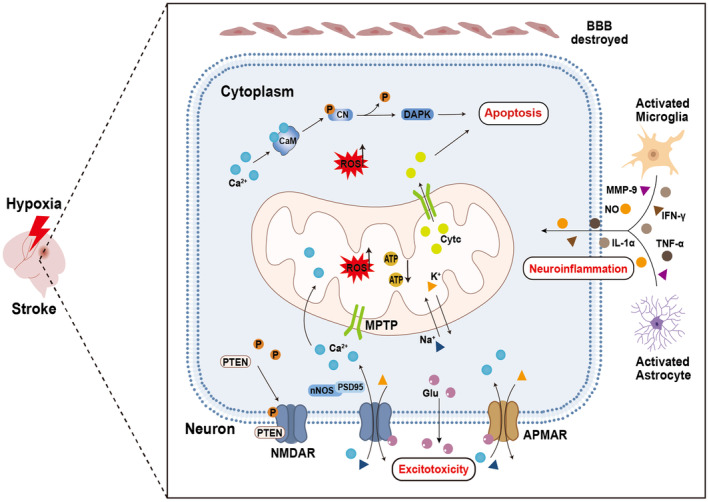
Pathophysiology due to hypoxia after stroke. Mitochondrial oxidative phosphorylation is inhibited, accumulating ROS and decreasing ATP synthesis. An insufficient supply of ATP leads to ion imbalance, depolarization of neurons and mitochondria, and Ca^2+^ influx. Neurons release glutamate and accumulate extracellularly, resulting in excessive activation of NMDARs and AMPARs, and PSD95 and nNOS bind to NMDARs, further promoting Ca^2+^ influx. Ca^2+^ influx leads to the opening of the mitochondrial MPTP and the release of Cytc, and Ca^2+^ activates calmodulin, stimulating calcineurin, which subsequently dephosphorylates and activates DAPK to induce apoptosis. Phosphatase and PTEN are recruited to NMDARs and induce neuronal death. The BBB is destroyed, and activated microglia and astrocytes produce and release many inflammatory mediators, which induce neuroinflammation.

After stroke, CBF is significantly reduced at the injury site, which limits the oxygen supply, especially to neurons. Mitochondrial oxidative phosphorylation is inhibited during hypoxia, resulting in reactive oxygen species (ROS) accumulation and ATP synthesis reduction (Figure 1).^22^ The ATP concentration and total adenine nucleotide content are significantly reduced, and glycogen is closely exhausted in the penumbra area in MCAO model rats.^23^ In hypoxic tissues, ADP generated from ATP hydrolysis is further metabolized to AMP and then converted to hypoxanthine and inosine, resulting in the overall depletion of adenine nucleotides.^24^

Hypoxia leads to mitochondrial dysfunction and oxidative stress‐induced injury.^25^ At the same time, energy deficiency also contributes to ion imbalances that change Na^+^, K^+^, and Ca^2+^ concentrations, leading to neuronal depolarization, glutamate release, and glutamate accumulation in the extracellular space.^26^ Excessive glutamate activates N‐methyl‐D‐aspartate receptors (NMDARs), inducing neurotoxicity, neuronal cell death, and ultimately CNS damage.^27^

### 3.2 Mitochondrial dysfunction, oxidative stress, and related signaling pathways

Mitochondria are essential for maintaining energy homeostasis. When ATP synthesis is blocked by hypoxia, the morphology and function of mitochondria are substantially altered. Ca^2+^ influx leads to mitochondrial permeability transition pore (MPTP) opening and cytochrome C (CytC) release (Figure 1).^28^ Moreover, an insufficient ATP supply triggers mitochondrial membrane depolarization, which is characterized by Na^+^ influx and K^+^ efflux.^29^, ^30^

Ferroptosis is a form of programmed cell death characterized by high intracellular iron accumulation and oxidative damage caused by iron‐dependent lipid peroxidation.^31^, ^32^, ^33^ Cell morphology is mainly characterized by a decrease in mitochondrial volume with a reduction of the condensed mitochondrial membrane density, the rupture of the mitochondrial outer membrane and the decline or disappearance of mitochondrial cristae, but the nucleus remains intact in structure without chromatin condensation.^34^, ^35^ The iron content in brain tissue increased, mediated ferroptosis, and exacerbated SBI in cerebral ischemia–reperfusion in a mouse model of MCAO.^36^, ^37^ Second, the iron released from the hematoma after ICH may lead to SBI.^38^, ^39^ Hemoglobin released from lysed red blood cells can be engulfed by macrophages and microglia around the hematoma and metabolized into Fe^2+^, which in turn mediates the production of ROS and lipid peroxidation.^40^, ^41^ Excess Fe^2+^ is transported from microglia and accumulates in neurons, forming hydroxyl radicals through the Fenton reaction. These radicals attack lipid membranes, proteins, and DNA, thereby destroying cellular function and promoting neuronal cell death.^42^, ^43^, ^44^

Cuproptosis is a recently defined form of cell death characterized by free copper accumulation and protein lipidation in mitochondria, leading to cytotoxic stress and, ultimately, cell death.^45^ This process depends on mitochondrial respiration and the tricarboxylic acid cycle. Copper directly binds to the lipid components of the tricarboxylic acid cycle, resulting in the aggregation of acylated proteins and the loss of iron–sulfur cluster proteins.^46^ The expression of proteins in the mitochondrial oxidative respiratory chain is reduced, and the activity of mitochondrial complexes I and III and ATP content are decreased, resulting in proteotoxic stress and cell death.^47^ Therefore, cuproptosis is speculated to be a key mechanism for the death of hypoxic–ischemic nerve cells.

Hypoxia can exacerbate oxidative stress. In addition to mitochondrial dysfunction, hypoxia leads to oxidative stress, which causes cellular and brain tissue injury after stroke.^48^ Oxidative stress disturbs the balance between ROS production and antioxidant defense. It plays a significant role in SBI after stroke. Considering the intimate connection between ROS and mitochondrial metabolism, mitochondrial dysfunction is often related to oxidative stress pathologies. During stroke hypoxia, oxidative damage and excessive Ca^2+^ contribute to MPTP induction, further promoting mitochondrial damage‐associated molecular patterns to activate downstream inflammatory responses.

### 3.3 Neuroinflammation

In the CNS, neurons are sensitive to energy and oxygen. Once neurons lack sufficient energy and oxygen, many pathological events will occur, including neuroinflammation. Cerebral hypoxia can cause damage to the brain microvascular system and blood–brain barrier (BBB), loss of tight junction proteins (TJPs), increased paracellular permeability, cytotoxic edema, and immune cell infiltration, thereby triggering an inflammatory response (Figure 1).^49^ Second, brain hypoxia activates inflammatory transcription factors and induces the synthesis and release of inflammatory mediators, cytokines, and enzymes.^50^, ^51^ Activated microglia and astrocytes produce and release many inflammatory mediators, such as TNF‐α, IL‐1α, IFN‐γ, ROS, nitric oxide (NO), and matrix metalloproteinase‐9 (MMP‐9), which further exacerbate the inflammatory response and brain injury.^49^, ^52^, ^53^, ^54^ In addition, hypoxia induces the expression of proinflammatory mediators (NO and TNF‐α) in microglia by regulating HIF‐1α.^55^, ^56^

### 3.4 Excitotoxicity and related signaling pathways

Typically, glutamate reuptake is an energy‐driven process. Under hypoxic conditions, ATP‐dependent neuronal physiological activities, such as ion transport and neurotransmitter reuptake, immediately decline. Hypoxia induces neuronal cell glutamate release after stroke. Glutamate then accumulates extracellularly, triggering excessive excitotoxicity events in neurons. The accumulated excitatory neurotransmitter glutamate overstimulates NMDARs and α‐amino‐3‐hydroxy‐5‐methyl‐4‐isoxazolepropionic acid receptors (AMPARs) and promotes a large influx of Ca^2+^ ions into the cell, promoting neuronal depolarization.^57^, ^58^ When neurons are depolarized, voltage‐gated Ca^2+^ channels and sodium channels are opened, allowing Ca^2+^ and Na^+^ to flow inward, where they are coupled with NMDARs and APMARs.^59^ Excessive Ca^2+^ influx perturbs ionic homeostasis, resulting in Ca^2+^ overload both in the mitochondria and cytosol.^60^, ^61^ This ion imbalance triggers the influx of water into neurons, causing edema and the dissolution of neuronal cells and tissue. Moreover, these changes stimulate a variety of proteases, lipases, kinases, phosphatases, endonucleases, and free radicals, as well as biological processes that cause neuronal cell death, such as calpain activation, oxidative stress, and mitochondrial impairment.^62^, ^63^ Overall, these cellular dysfunctions are termed excitotoxicity and involve NMDARs and AMPARs. The activation of NMDARs triggers neuronal excitotoxicity and subsequent apoptosis during ischemic stroke.^26^

This series of biological events caused by cerebral hypoxia leads to brain metabolic imbalance and constitutes the core pathological event of brain injury after stroke. The direct consequences are neuronal cell death and irreversible neurological injury. This stimulation also triggers multiple cascades and activates mitochondria‐dependent apoptosis after stroke.^61^ After NMDARs are activated by Ca^2+^ influx, phosphatase and tensin homolog (PTEN) is recruited to GluN2B‐NMDARs. PTEN signaling may induce neuronal cell death.^64^ As a Ca^2+^/calmodulin‐dependent serine/threonine‐protein kinase, dephosphorylated death‐associated protein kinase (DAPK) contributes to apoptotic cell death.^65^ After stroke, NMDAR overactivation promotes Ca^2+^ influx and calcineurin, dephosphorylating and activating DAPK (Figure 1). Both a selective NMDAR antagonist (MK‐801) and a calcineurin inhibitor (FK506) prevent the dephosphorylation of DAPK after ischemia, thereby exerting neuroprotective effects. These findings indicate that DAPK may be a potential therapeutic target for AIS.^66^ In addition, NMDAR‐mediated Ca^2+^ influx activates Ca^2+^‐dependent phosphatased calcineurin, rapidly increasing nuclear C/EBPβ and induces neuroexcitotoxicity.^67^ Neuronal NMDARs contribute to NO production, which is associated with Ca^2+^/calmodulin and regulated by NO synthase (nNOS). The binding of postsynaptic density protein 95 (PSD95) to NMDARs and nNOS enhances Ca^2+^ influx, a hallmark of excitotoxicity.^68^, ^69^ PSD95/nNOS signaling plays a pivotal role in ischemic stroke. Inhibiting nNOS activity ameliorates neurological deficits through pharmacological or genetic means in a cerebral ischemic model.^70^ Cerebral ischemia enhances NMDAR/PSD95/nNOS interactions in neurons, thus further aggravating brain injury in experimental ischemic stroke.^71^

## 4 UNDERLYING MECHANISMS OF NBO THERAPY AFTER STROKE

### 4.1 Rescue of tissue hypoxia and metabolism

**FIGURE 2:**
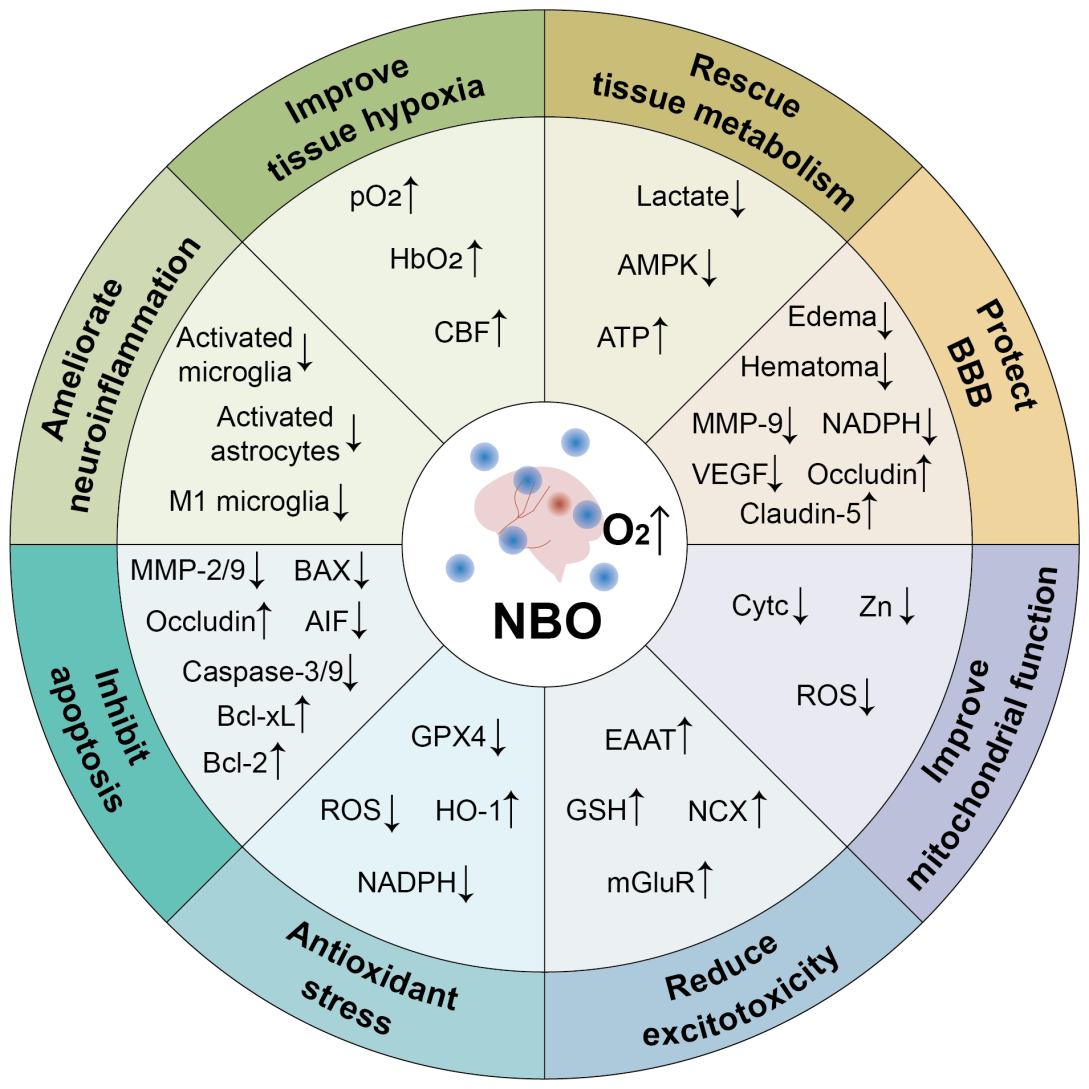
Molecular mechanisms of NBO treatment. Various critical molecules and mechanisms are involved in the neuroprotective effects of NBO treatment. AIF, apoptosis‐inducing factor; AMPK, AMP‐activated protein kinase; ATP, adenosine 5′‐triphosphate; BAX, Bcl2‐associated X; Bcl‐2, B‐cell lymphoma‐2; Bcl‐Xl, B‐cell lymphoma‐extra‐large; CBF, cerebral blood flow; Cytc, cytochrome C; EAAT, glutamate transporters; GPX4, glutathione peroxidase 4; GSH, glutathione; HbO_2_, oxyhemoglobin; HO‐1, heme oxygenase‐1; mGluR, glutamate receptors; MMP‐2/9, matrix metalloproteinase‐2/9; NADPH, triphosphopyridine nucleotide; NCX, Na^+^/Ca^2+^ exchanger; pO_2_, partial oxygen; ROS, reactive oxygen species; VEGE, vascular endothelial growth factor; Zn, zinc.

AIS results in marked heterogeneous changes in tissue oxygenation, with severe hypoperfusion in the core and a moderate reduction in blood flow in the penumbra.^72^ Using electron paramagnetic resonance oximetry to measure localized interstitial partial oxygen (pO_2_), Liu et al.^73^ reported that interstitial pO_2_ rapidly decreased to approximately 4% in the core and 30% of preischemic values in the penumbra in a rat cerebral ischemia model. They further demonstrated that 95% of NBO administration maintained penumbral interstitial pO_2_ levels close to preischemic levels during ischemia and twofold increased pO_2_ levels during reperfusion (Figure 2).^74^ Oxygen is a vital substrate that supports virtually all metabolic processes. In a rat model of distal MCAO, NBO (100% O_2_) administration caused an immediate and progressive increase in oxyhemoglobin concentration, nearly doubling in the ischemic core within 1 h, preserved CBF in the core and penumbra, suppressed peri‐infarct size and depolarization and diminished the deleterious effects on CBF and metabolic load.^75^

Under hypoxic conditions, glucose metabolism in the brain switches to anaerobic glycolysis, drastically reducing ATP production.^76^ Metabolic abnormalities are another major contributor to brain injury after stroke. NBO has been shown to reduce ATP consumption in the marginal zone tissue and acidosis in focal cerebral ischemia.^77^ Using multivoxel magnetic resonance spectroscopic imaging, lactate, a marker of aerobic metabolism, decreased during NBO administration via inhalation of oxygen at a flow rate of 45 L/min through a face mask for 8 h in stroke patients.^78^ The penumbra can be rescued if blood flow is restored in time.^58^, ^79^ NBO can be used as an early intervention to preserve the penumbra and expand the window of reperfusion therapy.^80^ In a Sprague–Dawley rat model of transient MCAO and permanent ischemia, Ding et al.^81^ demonstrated that ethanol combined with NBO administration (95% O_2_) significantly attenuated the expression of monocarboxylate transporters and lactate levels. In a rat model of permanent MCAO, NBO therapy significantly increased ATP production, which was associated with reduced transcriptional and translational levels of the hyperglycolysis enzymes glucose transporter 1 and 3, phosphofructokinase 1, and lactate dehydrogenase. In addition, NBO significantly decreased AMPK mRNA and protein expression levels.^82^ The occurrence of stroke first leads to ischemia and hypoxia in tissues, which in turn affects the metabolism of tissues and the occurrence of a series of pathological events. NBO therapy effectively provides tissue support and rescues tissue hypoxia and metabolism after stroke, thereby reducing the occurrence of subsequent pathological events.

### 4.2 Protecting against BBB impairment and ameliorating brain edema

Fujiwara et al.^83^ reported that applying 100% NBO for 2 h did not worsen hemorrhage severity, brain edema, or neurological outcomes on Day 3 in a collagenase type VII‐induced ICH model in Sprague–Dawley rats. Gaberel et al.^84^ demonstrated that NBO treatment for 30 min induced rapid and progressive reduction of the apparent hematoma on longitudinal T2*‐weighted images in a collagenase‐induced and direct intrastriatal injection blood ICH model in Swiss mice. NBO therapy (90% O_2_) for 6 h daily for three consecutive days improved the neurological severity score (NSS), attenuated brain edema and cellular apoptosis, and suppressed HIF‐1α and VEGF in the perihematomal region in a collagenase‐induced ICH model in Sprague–Dawley rats.^85^ NBO or HBO at three atmospheres initiated within 30 min for 1 h attenuated brain edema and BBB disruption in a mouse model of autologous blood‐ or collagenase‐induced ICH. Early oxygen therapy attenuated posthemorrhagic occludin degradation, MMP‐9 activation, and HIF‐1α expression (Figure 2).^86^ In addition, NBO treatment rescued microglia‐mediated synaptic pruning in collagenase‐induced ICH in mice. NBO therapy effectively restored postsynaptic protein (PSD and NR2B) expression levels and improved the loss of synaptic spines, which play a neuroprotective role after ICH.^87^

Liang et al.^88^ revealed that NBO treatment (100% O_2_) for 30 min slowed BBB damage, as evidenced by reduced Evan blue leakage, edema, and hemorrhagic volume at 3, 5, and 7 h in a rat model of MCAO and reperfusion (MCAO/R). NBO‐mediated protection of the BBB was sustained during tissue‐type plasminogen activator (t‐PA) administration, which revealed that NBO may be an effective adjuvant for extending the time window for t‐PA thrombolysis. NBO (100% O2) treatment significantly reduced BBB damage by reducing the expression of MMP‐9, the loss of TJPs, and the degradation of occludin and claudin‐5 in ischemic cerebral microvessels, thereby improving the neurological function of rats with MCAO/R and stroke patients.^88^, ^89^ NBO (95% O_2_) treatment inhibited NADPH oxidase activity and MMP‐9 induction, which has been demonstrated to protect against BBB and brain edema in a rat model of MCAO.^90^, ^91^ Yang et al.^92^ revealed that exposure to 60% and 100% NBO during reperfusion following MCAO improved neurological impairment scores and alleviated brain edema, neutrophil infiltration and infarct volume after 24 and 48 h. Moreover, 100% NBO further improved the parameters.^93^ In addition, the molecular mechanisms by which NBO reduces brain edema may involve a reduction in aquaporin 4 and Na^+^/H^+^ exchanger 1 and an increase in HIF‐1α expression.^92^ NBO (40% O_2_) administration significantly improved blood perfusion in the ischemic area early and effectively alleviated BBB permeability, cerebral edema, and neurological dysfunction in MCAO model mice.^94^

In contrast, Elmira Pasban reported that early NBO administration for 90 min did not reduce ischemic brain injury and exerted protective effects against vasogenic brain edema formation or BBB disruption in adult male Sprague–Dawley rats subjected to MCAO for 90 min followed by 24 h of reperfusion.^95^ However, in general, NBO treatment after stroke can reduce brain edema and BBB permeability and play a protective role in the BBB.

### 4.3 Ameliorating the neuroinflammatory response

Esposito et al.^96^ revealed that the protective effect of NBO persists for up to 2 weeks by suppressing microglia and astrocyte reactions and reducing infarct volume in a rat model of transient MCAO (Figure 2). NBO administration alleviated brain damage and sensorimotor impairment by preventing neuronal cell death and microglial inflammation in a rat model that mimics a true transient ischemic attack.^97^ In addition, HBO pretreatment downregulated the JNK/STAT pathway, attenuated M1 microglial polarization, and reduced the expression of proinflammatory factors and MMP‐9, thereby alleviating neuroinflammation and reducing ICH‐induced neurological damage and brain edema in an autologous blood‐induced model of ICH in rats.^98^

### 4.4 Improving mitochondrial function and antioxidant stress

NBO administration during 2 h of MCAO and 1 h of reperfusion reduced the infarct volume without increasing oxidative stress through the expression of heme oxygenase‐1 (HO‐1) and protein carbonyl formation (Figure 2).^99^ NBO treatment maintained the homeostasis of glutathione peroxidase 4 (GPX4) and NADPH oxidase 4 oxidoreductases and rescued MCAO/R‐induced downregulation of connexin 43 protein in astrocytes. In addition, NBO treatment attenuated ischemia‐induced mitochondrial CytC release, which could be blocked by inhibiting the GPX4 or connexin 43 pathway.^100^ Dong et al.^101^ reported that NBO therapy significantly reduced zinc accumulation and mitochondrial CytC release and stabilized the mitochondrial membrane potential in penumbra tissue. Still, the zinc content in the ischemic core area did not decrease in a rat model of MCAO. These findings indicated that NBO administration prevented ischemia‐induced mitochondrial zinc accumulation, thereby improving mitochondrial function. Compared with control treatment, NBO treatment significantly decreased ROS levels at 3 and 24 h after reperfusion.^82^ NBO treatment inhibited NADPH oxidase activity and reduced ROS production, thereby alleviating oxidative stress caused by ischemia and protecting the brain in a rat model of MCAO.^102^, ^103^ Compared with any treatment alone, 95% NBO and ethanol (EtOH) combination therapy effectively suppressed the ADP/ATP ratio, ROS generation, and nicotinamide adenine dinucleotide phosphate oxidase (NOX) activity and maximized pyruvate dehydrogenase (PDH) activation and protein expression, which exerted a synergistic neuroprotective effect after reperfusion.^104^ Cai et al.^105^ revealed that following t‐PA in a rat model of focal middle cerebral artery (MCA) stroke, NBO (60% O_2_) combined with hypothermia (Hypo) or EtOH effectively decreased neurological deficit and infarct volume, as well as ROS production, compared with monotherapies, this finding suggested that the PKC‐Akt‐NOX pathway is involved in the mechanism of antioxidative injury.

The survival of neurons largely depends on the energy provided by mitochondria.^106^ Mitochondrial dysfunction after stroke not only reduces ATP production but also produces a large amount of ROS and increases oxidative stress. After stroke, NBO treatment can effectively ameliorate mitochondrial dysfunction and reduce oxidative stress, thus playing a neuroprotective role.

### 4.5 Reducing calcium overload and neuroexcitotoxicity

After prolonged or intermittent NBO preconditioning in a rat model of MCAO, the neurologic deficit score significantly decreased, and the serum TNF‐α level and glutamate transporter (EAAT1, EAAT2, and EAAT3) expression levels in brain tissue significantly increased, which induced cerebral ischemic tolerance in rats (Figure 2).^107^ Nasrniya et al.^108^ also showed that NBO induced cerebral ischemic tolerance in rats. Intermittent NBO pretreatment reduced the infarct volume and neurologic deficit score, increased the glutathione level in the subcortex region, and significantly upregulated the expression of metabotropic glutamate receptors (mGluRs I and II) in the MCAO model rats. The expression of the Na^+^/Ca^2+^ exchanger (NCX) was increased after NBO pretreatment in MCAO model rats. The expression of NCX2 and NCX3 in the core, NCX1, NCX2, and NCX3 in the penumbra, and NCX1 and NCX3 in the subcortex are increased.^109^ In addition, Haelewyn et al.^110^ demonstrated that NBO attenuated NMDA‐induced neuronal degeneration in rats and reduced NMDA‐induced Ca^2+^ influx in cultured neuronal cells. However, NBO increased ischemia‐induced injury in a rat MCAO model and an oxygen–glucose deprivation (OGD) neuronal model.

Hypoxia after stroke induces glutamate release from neurons and extracellular accumulation, which in turn triggers excitotoxic events. Moreover, the opening of Ca^2+^ channels lead to the influx of Ca^2+^, which overloads Ca^2+^ in the cytoplasm and mitochondria and further exacerbates excitotoxicity. NBO treatment after stroke regulates the expression of glutamate transporters, metabotropic glutamate receptors and NCX, reduces the influx of Ca^2+^ and neuroexcitotoxicity, and plays a neuroprotective role.

### 4.6 Inhibiting neuronal cell death

Apoptosis plays an important role in neuronal cell death after stroke. NBO combined with minocycline effectively reduced transient focal cerebral ischemia‐induced brain injury in a rat model of MCAO. Compared with monotherapy, combination therapy more effectively inhibited the expression of MMP‐2/9, the degradation of occludin, the activation of caspase‐3/9 and the expression of apoptotic‐inducing factor (AIF) in ischemic brain tissue (Figure 2).^111^ Geng et al.^112^ suggested that in a rat model of MCAO, NBO combined with ethanol significantly reduced the expression of proapoptotic proteins (BAX, caspase‐3, and AIF) and increased the expression of antiapoptotic factors (Bcl‐2 and Bcl‐xL). This intervention reduced the percentage of apoptotic cells by 49%, thereby protecting neurological function. NBO administration started after 30 min of ischemia in rats and lasted for 2, 4, or 8 h. NBO treatment for 8 h more effectively reduced infarct volume and oxidative stress than other treatment durations. Moreover, DNA fragment generation and caspase‐3 activity in the cortical penumbra were reduced, effectively reducing apoptosis.^103^ By combining EtOH or Hypo with t‐PA, 60% NBO enhanced neuronal antiapoptotic effects by reducing the expression of the proapoptotic protein AIF, activated caspase‐3, and Bax and increasing the expression of the antiapoptotic protein Bcl‐2.^113^ In addition, prolonged NBO treatment during ischemia and the early phase of reperfusion alleviated neuronal apoptosis by inhibiting the MMP‐2/PARP‐1 pathway in MCAO model rats.^114^

Moreover, Wang et al.^115^ reported that NBO administration not only reduced neuronal apoptosis but also alleviated autophagy in the early stages of reperfusion after ischemia/reperfusion. LC3 and Beclin1 were significantly decreased, and p63 was significantly increased at 2 h and 6 h of reperfusion. In conclusion, NBO treatment effectively reduces stroke‐induced apoptosis and protects neurological function after stroke. Notably, NBO treatment can effectively reduce the generation of ROS, inhibit oxidative stress, reduce the release of Cytc from mitochondria, and ameliorate mitochondrial dysfunction after stroke.^82^, ^101^ Thus, NBO treatment may reduce the production of ROS and hydroxyl radicals by inhibiting oxidative stress, reducing the oxidative damage caused by lipid peroxidation, and increasing the expression of related proteins or enzymes in mitochondria. These changes affect ferroptosis and cuproptosis and thereby improve mitochondrial dysfunction and patient prognosis. However, definite evidence for this mechanism is lacking, and this mechanism warrants further study.

## 5 CLINICAL EVIDENCE

NBO may freeze the penumbra and extend the time window for reperfusion, making it an ideal adjunct for endovascular thrombectomy.^80^, ^116^ The role of NBO in clinical trials is summarized in Table 1.

**TABLE 1:** The effect of NBO in clinical trials.

Subjects	Sample size	Treatment and duration	Outcome	References
Patients with acute stroke and perfusion‐diffusion “mismatch” on MRI	16	100% O_2_, 45 L/min, 8 h, by simple facemask	DWI lesion volume and NIHSS score decreased	[112]
Patients experiencing a first‐ever massive middle cerebral artery infarction	46	40% O_2_, 2 L/min, 132.9 (range, 48.0–168.5) h, by venturi mask	Mortality and complications decreased	[113]
Patients with acute stroke and perfusion‐diffusion “mismatch” on MRI	6	100% O_2_, 45 L/min, 8 h, by simple facemask	N‐acetylaspartate retained and lactate reduced in the brain	[73]
Patients with ICA and/or proximal MCA occlusion and a diffusion/perfusion mismatch at presentation	14	100% O_2_, 45 L/min, 8 h, by simple facemask	A stable mismatch of 4 h or longer occurred outside the time window of thrombolytic therapy	[114]
Patients with acute stroke and perfusion‐diffusion “mismatch” on MRI	19	100% O_2_, 45 L/min, 8 h, by simple facemask	Ischemic lesions growth was attenuated	[115]
Patients with AIS	231	100% O_2_, 10 L/min, 4 h, by simple facemask, endovascular treatment	The infarct volume was reduced, and the incidence of symptomatic intracranial hemorrhagic, mortality, and adverse events were decreased	[117]
Patients with anterior circulation stroke	175	50% FiO_2_, 15 L/min, 6 h, by venturi mask, mechanical thrombectomy	The infarct volume was reduced, and the 90‐day mortality was reduced	[118]
Patients with posterior circulation stroke	87	50% FiO_2_, 15 L/min, 6 h, by venturi mask, mechanical thrombectomy	The infarct volume was reduced, and the 90‐day mortality was reduced	[119]
Patients with AIS	40	100% O_2_, 10 L/min, 12 h, by simple facemask	Clinical score (NIHSS, mRS, and BI) no difference	[120]

NBO therapy was safe and feasible in an initial clinical trial and was associated with transient improvements in clinical deficits and MRI lesion volumes at 4 h in AIS patients. The mean relative cerebral blood volume (CBV) and CBF increased significantly at 4 h and 24 h in the NBO group.^117^ Chiu et al.^118^ used 40% oxygen inhalation via a venturi mask to treat patients with severe AIS. Compared with nasal cannula oxygen therapy, venturi masks are associated with lower mortality and fewer comorbidities in patients with severe AIS. Singhal et al.^78^ monitored the signal levels of lactic acid and N‐acetylaspartate using the software package LCModel and found that NBO treatment retained N‐acetylaspartate and reduced lactate levels in the brains of AIS patients. This finding suggested that NBO can improve aerobic metabolism in the ischemic brain and preserve neuronal integrity. Subsequent trials by Singhal's group further demonstrated the safety and efficacy of NBO. Compared with controls, patients outside the thrombolytic treatment time window experienced greater diffusion/perfusion mismatch on MRI 4 h or more after NBO treatment.^119^ Wu et al.^120^ also showed that 4 h of NBO therapy can reduce the growth of ischemic lesions. In addition, randomized controlled trial protocols are being explored for the efficacy and safety of NBO in patients with acute ICH.^121^

According to several recent clinical reports, NBO combined with endovascular thrombectomy seems to be a safe and feasible treatment for AIS. Li et al.^122^ reported that compared with EVT, NBO + EVT can significantly reduce the infarct volume, improve the prognosis, and decrease the incidence of symptomatic intracranial hemorrhage, mortality, and adverse events, but these effects are not statistically significant. In addition, Cheng et al.^123^ reported that adjuvant high‐flow NBO treatment after mechanical thrombectomy for anterior circulation stroke can significantly reduce the infarct volume and 90‐day mortality rate and effectively improve the clinical outcome of patients with anterior circulation stroke within 6 h of onset. Similarly, they performed the same experiment in posterior circulation stroke patients. Infarct volume and 90‐day mortality were reduced, but this difference was not statistically significant.^124^ Conversely, a clinical trial has also shown that NBO has no effect. Padma et al.^125^ conducted NBO treatment on Indian AIS patients and analyzed a series of clinical scores (NIHSS, mRS, and BI), which showed that NBO did not improve the clinical outcomes of stroke patients.

## 6 POTENTIAL ADVERSE EFFECTS OF NBO ON STROKE OUTCOMES

NBO therapy not only exerts protective effects on the brain but also may produce adverse effects, such as oxygen toxicity, cerebral vasoconstriction, neuroexcitotoxicity, and lung injury. Quintard et al.^126^ showed that NBO treatment increased the glutamate content in the brains of patients with severe traumatic brain injury, causing brain excitotoxicity and exacerbating SBI. A high oxygen supply to the brain is well known to potentially cause cerebral vasoconstriction, which results in insufficient cerebral hypoperfusion. This change can increase intracranial pressure, enlarge brain hematomas and edema, deteriorate neurological function and have other negative effects.^127^, ^128^ High concentrations of oxygen can easily cause oxygen toxicity.^129^ Alva et al.^130^ summarized the mechanism of NBO‐induced oxygen toxicity and discussed strategies to improve the damage caused by hyperoxia. The formation of ROS is the biochemical basis of oxygen toxicity and one of the causes of SBI.^131^ When excessively high concentrations of oxygen accumulate in brain tissue, ROS levels in neuronal cells further increase, thus affecting the signaling pathway in mitochondria and activating the release of CytC, which leads to cell death and accelerates the progression of tissue damage.^132^ In addition, ROS can cause lipid peroxidation, protein oxidation, and DNA damage.^133^, ^134^, ^135^ However, ROS can also increase the activity or expression of antioxidant enzymes in cells to remove excessive ROS.^136^, ^137^ Whether NBO treatment leads to an increase in ROS, which can cause damage to neurons, remains debated. At present, the generally accepted view for treating AIS is that the benefits of NBO treatment significantly outweigh the potential risk of ROS.^137^, ^138^ Second, oxygen toxicity may exacerbate lung injury and cause pulmonary hypertension and pulmonary fibrosis.^139^ High concentrations of oxygen enter the lungs, replacing the nitrogen present in the alveoli and rapidly diffusing into the plasma, causing the alveoli to collapse and reducing alveolar volume.^140^ In addition, because human organs (heart, digestive tract, eyes, etc.) are sensitive to high concentrations of oxygen, NBO may cause symptoms such as angina, nausea and vomiting, and lens degeneration.^141^, ^142^

To reduce potential adverse reactions to NBO treatment, the oxygen concentration and oxygen inhalation time should be controlled, and brain tissue oxygen monitoring or cerebral microdialysis should be performed. Controlling NBO (100% O_2_, 10 L/min) treatment for 4–6 h may increase the effectiveness of treatment for patients with acute ischemic stroke.^143^ Cerebral microdialysis technology can continuously and dynamically monitor the levels of substances closely related to oxygen therapy in the brain, such as glucose, lactic acid, ROS‐related substances, neurotransmitters, and brain injury markers.^144^ Before oxygen inhalation, the solution should be heated and humidified first, and atomized inhalation should be regularly administered to reduce the stimulation effect.^145^ Patients should be encouraged to take deep breaths, cough more and change positions frequently to prevent secretions from blocking the respiratory tract. NBO has potential protective effects in the treatment of stroke, but strategies to avoid the occurrence of adverse reactions and maximize the effect of NBO warrant further study.

## 7 CONCLUSIONS AND PERSPECTIVES

Oxygen is essential for sustaining life. Stroke causes insufficient blood and oxygen supply to the brain, triggering a series of pathological injuries, including mitochondrial dysfunction, energy deficiency, oxidative stress, neuroinflammation, and excitotoxicity. Oxygen therapy can effectively slow the occurrence of pathological events and prevent further damage to the brain. This review summarizes the effects and related mechanisms of NBO therapy after stroke, including rescuing tissue hypoxia, protecting the BBB, reducing brain edema and neuroinflammation, improving mitochondrial dysfunction, resisting oxidative stress, and reducing excitotoxicity and apoptosis, this review also summarizes the role of NBO in clinical trials. NBO is a simple and noninvasive therapy with marked advantages and can be used for early stroke treatment. However, controversies and challenges regarding the application of oxygen therapy in stroke patients persist. For example, which patients are suitable for oxygen therapy? When is the best time for oxygen therapy? How can the concentration, time, and method of oxygen therapy be determined? These issues need to be further explored. Therefore, we look forward to more studies that can evaluate the role of oxygen therapy in stroke treatment to provide more accurate and useful information for clinical practice, more treatment options, and better prognosis for stroke patients.

## AUTHOR CONTRIBUTIONS

The initial idea for this review was conceived by M.X.W., J.W., X.P.Y., and C.L., and the manuscript was written and revised by C.L., M.X.W., M.J., and Z.Y.C. In addition, Z.Y.C., Q.Q.H., Z.Y.L., and J.M.W. contributed to the preparation of the illustrations; J.W. and M.J. edited the manuscript; and C.L., J.W., and M.X.W. read, reviewed, and approved the final manuscript. All the authors approved the final version of the manuscript.

## FUNDING INFORMATION

This study was partially supported by the National Natural Science Foundation of China (82260209 and 81960221 to XPY; 82371339 to JW), the Jiangxi Provincial Natural Science Foundation of China (20232BAB206046 to MXW), the Science and Technology Project Founded by the Education Department of Jiangxi Province (GJJ201834 to MXW), the Jiangxi Provincial Health Commission Science and Technology Plan project (202212021 to MXW), and the Natural Science Foundation of Henan Province for Young Scholars (242300421515 to QQH).

## CONFLICT OF INTEREST STATEMENT

Jian Wang is an editorial board member of CNS Neuroscience and Therapeutics and a coauthor of this article. To minimize bias, he was excluded from all editorial decision‐making related to the acceptance of this article for publication. The other authors declare that they have no conflicts of interest.

## Data Availability

Data sharing not applicable to this article as no datasets were generated or analysed during the current study.
